# Research on a Method to Improve the Temperature Performance of an All-Silicon Accelerometer

**DOI:** 10.3390/mi14040869

**Published:** 2023-04-18

**Authors:** Guowen Liu, Yu Liu, Xiao Ma, Xuefeng Wang, Xudong Zheng, Zhonghe Jin

**Affiliations:** 1 School of Aeronautics and Astronautics, Zhejiang University, Hangzhou 310058, China; 2 Beijing Institute of Aerospace Control Device, Beijing 100854, China

**Keywords:** MEMS accelerometer, response signal, anchor zone, stress cancellation

## Abstract

This paper presents a novel method for the performance of an all-silicon accelerometer by adjusting the ratio of the Si-SiO_2_ bonding area, and the Au-Si bonding area in the anchor zone, with the aim of eliminating stress in the anchor region. The study includes the development of an accelerometer model and simulation analysis which demonstrates the stress maps of the accelerometer under different anchor–area ratios, which have a strong impact on the performance of the accelerometer. In practical applications, the deformation of the comb structure fixed by the anchor zone is influenced by the stress in the anchor region, causing a distorted nonlinear response signal. The simulation results demonstrate that when the area ratio of the Si-SiO_2_ anchor zone to the Au-Si anchor zone decreases to 0.5, the stress in the anchor zone decreases significantly. Experimental results reveal that the full-temperature stability of zero-bias is optimized from 133 μg to 46 μg when the anchor–zone ratio of the accelerometer decreases from 0.8 to 0.5. At the same time, the full-temperature stability of the scale factor is optimized from 87 ppm to 32 ppm. Furthermore, zero-bias full-temperature stability and scale factor full-temperature stability are improved by 34.6% and 36.8%, respectively.

## 1. Introduction

Accelerometers are widely used in various areas, including navigation, aviation, aerospace, weapons, and civil fields. However, the traditional accelerometer’s large size and high cost limits its applications. With the development of microelectromechanical systems (MEMS) technology, various MEMS accelerometers have emerged. Its characteristics of small size, low-power consumption, and wide application range have aroused the research interest of all circles [1]. N. Yazdi introduced an all-silicon structure accelerometer in approximately 2000 [2]. All-silicon structure accelerometers have the advantage of low temperature sensitivity and good long-term stability due to their material consistency. Colibrys, a division of Safran, introduced a new structure of the all-silicon sandwich accelerometer in 2020 [3], which improved the zero-bias full temperature stability to 30 μg. In 2016, D. Xiao reported a dual-differential torsional MEMS glass–silicon–glass sandwich accelerometer structure, in which the characteristics of the temperature coefficient were five times lower than before [4]. In 2018, Wei Xu reported an all-silicon structure of the dual-differential accelerometer, in which the total temperature stability was increased by three times [5]. In 2020, H. Niu reported that the same type of accelerometers had a zero-bias stability of 100 μg [6]. In 2018, Huan Liu reported two versions of capacitive accelerometers based on low-temperature co-fired ceramic (LTCC) technology, presenting a larger full-scale range (10 g), and lower nonlinearity of less than 1%, as well as a sensitivity of 30.27 mV/g [7]. The MEMS Technology Center at the Middle East University of Science and Technology in Turkey has manufactured a Silicon-On-Insulator (SOI) structure of the triaxial capacitive accelerometer, which had a background noise of 8 μg/√Hz, as recorded in 2020 [8]. In 2021, Yurong He reported a novel teeter-totter type accelerometer based on glass–silicon composite wafers, in which a zero-bias stability under 0.2 mg, and a noise floor with 11.28 μg/√Hz were obtained [9]. In 2022, Yongjun Zhou reported an improved variational mode decomposition (VMD), and the time-frequency peak filtering (TFPF) denoising method has a smaller amount of signal distortion and a stronger denoising ability, so it can be adopted to denoise the output signal of the High-G MEMS accelerometer in order to improve its accuracy [10]. Litton SiAC^TM^ has reported a silicon-based MEMS accelerometer made of an all-silicon structure, with a measurement range of over 100 g, it has good characteristics including zero-bias stability and a scale factor stability that is better than 20 μg and 50 ppm.

The working mechanism of the accelerometer is based on Newton’s law of inertia. It is a mechanical sensitive device, so all kinds of stresses will bring the output error of the accelerometer and deteriorate the full-temperature performance of the accelerometer. At present, there are three main measures to reduce the stress effect for accelerometers. First, stress isolation can be achieved by adding a stress isolation frame and stress isolation beam, or by low-stress encapsulation [11,12,13]. Second, the effect of in-plane stress is reduced by stress difference using differential symmetry structures [14]. Third, an all-silicon wafer level packaging process can be used to reduce the thermal stress caused by inconsistent coefficients of thermal expansion between layers [15]. The all-silicon wafer level packaging process can reduce the thermal stress caused by the inconsistent thermal expansion coefficients of the cap layer, the sensitive structure layer, and the substrate layer. However, the stress isolation structure will increase the acceleration sensor volume. Although the above low-stress packaging technology can reduce the external stress interference, it cannot reduce the internal stress of the chip. The differential symmetric structure mainly reduces the stress by the symmetric structure in X, Y, and Z directions. In this paper, based on the above stress-reduction technology, the internal stress reduction is further studied to adapt to the complex multi-layer structure of the MEMS accelerometer. Therefore, a bonding anchor zone stress cancellation method is proposed to reduce thermal stress transferred to the sensitive capacitor, which improves the accelerometer’s overall temperature performance. This study provides a theoretical basis for the development of high-precision capacitive MEMS accelerometers.

## 2. MEMS Accelerometer Layer Design

**Figure 1:**
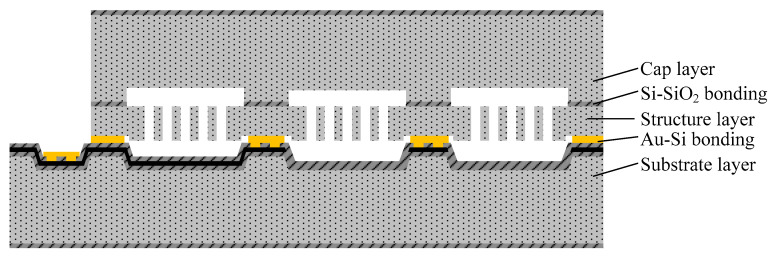
Structural Diagram of MEMS Accelerometer.

Figure 1 shows the chip layer design of an all-silicon accelerometer, which consists of three layers: substrate layer, sensitive structure layer, and cap layer. A silicon dioxide graphic layer is present between the cap layer and the structure layer. The structure layer is bonded to the cap layer using Si-SiO_2_ bonding, while the gold-silicon eutectic bonding is used to bond the sensitive structure layer to the substrate layer. This bonding arrangement creates a cavity in which the micro-structure can move freely. Electrodes are patterned on the substrate layer, and a coplanar electrode is used to connect the sensitive structure inside the cavity with the outer electrode pad. The anchor area is the bonding area between the cap layer and the sensitive structure layer, connecting the three layers together and supports the movable sensitive structure.

## 3. Principle of MEMS Accelerometer

**Figure 2:**
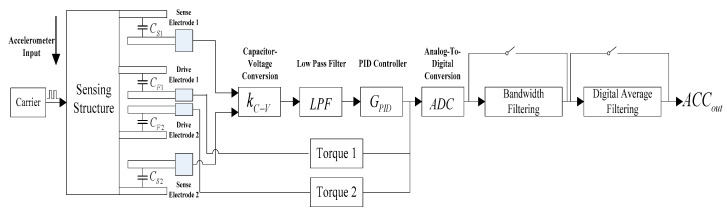
Principle block diagram of MEMS accelerometer.

As shown in Figure 2, the MEMS accelerometer system consists of several components, including accelerometer-sensitive structure, capacitance-voltage conversion module, low-pass filter (LPF), proportional integral derivative (PID) controller, torquer, analog-to-digital conversion module (ADC), digital bandwidth filtering module, and digital average filtering module. Under external input acceleration, the accelerometer sensitive structure moves relative to the carrier coordinate system. The distance between the sense electrode 1 and the movable comb electrode increases, so the sensing capacitance C_S1_ decreases, while the distance between the sense electrode 2 and the movable comb electrode decreases, the sensing capacitance C_S2_ increases. The sensing capacitance C_S1_ and the sensing capacitance C_S2_ are converted into two voltage values through a capacitor-voltage conversion circuit (CV), and then converted to differential voltage, which is sampled by the analog-to-digital converter module after passing through a low-pass filter and PID controller. After that, it is then passed through a bandpass filter and digital average filter to work as the output signal of the accelerometer. The output of the PID controller is amplified by the torque 1 and torque 2 circuits and fed back to the drive electrode 1 and drive electrode 2 of the accelerometer, respectively. The drive electrode 1 and the movable comb electrode form the drive capacitor C_F1_, and the drive electrode 2 and the movable comb electrode form the drive capacitor C_F2_. The electrostatic attraction of C_F1_ is greater than that of C_F2_ due to the different voltages applied by the torques to the two drive capacitors, which keeps the accelerometer-sensitive structure static near the initial position and thus forms an electrostatic balance closed-loop system.

## 4. Structural Stress Simulation Analysis of MEMS Accelerometer

The comb-tooth capacitive MEMS accelerometer described in this paper has a symmetrical arrangement of its sensitive structure layer. However, the cap layer and substrate layer above and below the structure layer are not completely symmetric. The anchor layer between the cap layer and the structure layer is made of silicon dioxide, while the anchor layer supported by the substrate layer and the sensitive structure layer is made of gold. It can be seen from the material properties in Table 1 that the thermal expansion coefficients of these materials are inconsistent, causing different stresses on the anchor area on both sides of the structure at different temperatures, which reduces the accelerometer’s temperature performance. To address this issue, a stress cancellation method in the anchor zone is proposed in this paper. The optimal stress of the sensitive structure over full-temperature can be achieved by matching the anchor area of the upper and lower sides of the sensitive structure to achieve the balance of stress on both sides. The asymmetric stress is reduced drastically, and thus the full-temperature accuracy of the MEMS accelerometer is improved.

The stress generated by the bonding of the MEMS accelerometer is related to the anchor zone, and the balance of the stress on both sides of the structure layer is achieved by designing the size of the anchor zone on both sides of the sensitive structure layer, so that the stress of the sensitive structure is optimized at full-temperature, and the generation of asymmetric stress is fundamentally reduced, thereby improving the full-temperature accuracy of the accelerometer. Since the normal force and the lateral force are related to Poisson’s ratio of the material, the lateral force has a strong correlation with the normal force, so optimizing the normal force also indirectly optimizes the lateral force, which may obtain a similar result in this research. Therefore, only the normal force is considered as the research object in this paper. The positive stress is multiplied by the area of the anchor area in order to obtain the stress value, and the stress on both sides of the anchor zone is treated with a combined force, and the point with the smallest resultant force is the most advantageous. By simulating the force curves of different anchor–zone ratios, the optimized result is selected and tested. The design of the acceleration is only different in the anchor zone, and the processing is consistent, so as to minimize external interference factors as much as possible. Finally, the change in the output result is strongly correlated with the change in the anchor–zone ratios, in order to complete the experimental verification.

**Figure 3:**
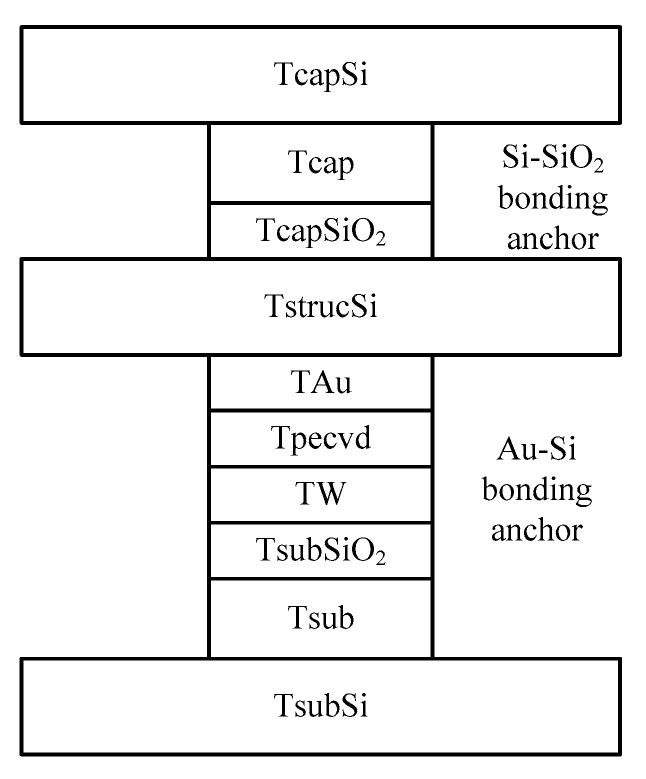
Anchorage simulation modeling model.

Since the in-plane structure is fully symmetric, each anchor area has the same size. During simulation, a group of anchor areas are taken for modeling, as shown in Figure 3. The model consists of 10 layers, and the upper and lower layers of the structure are the silicon dioxide anchor layer and the gold silicon anchor layer, respectively.

**Figure 4:**
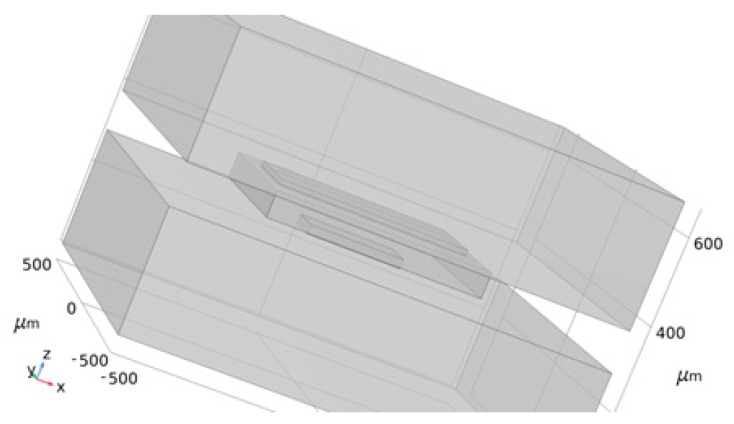
Anchor zone simulation diagram.

Figure 4 shows the simulation diagram of the stress model of the anchor zone, where the stress difference of the anchor zone is the added stress difference of the Si-SiO_2_ zone and the Au-Si zone at different temperatures. The force on the anchor zone is the surface normal stress multiplied by the area of the anchor zone. The anchor–zone ratio is defined as the square root of the ratio of the area of the Si-SiO_2_ anchor zone divided by the area of the Au-Si anchor zone.

**Figure 5:**
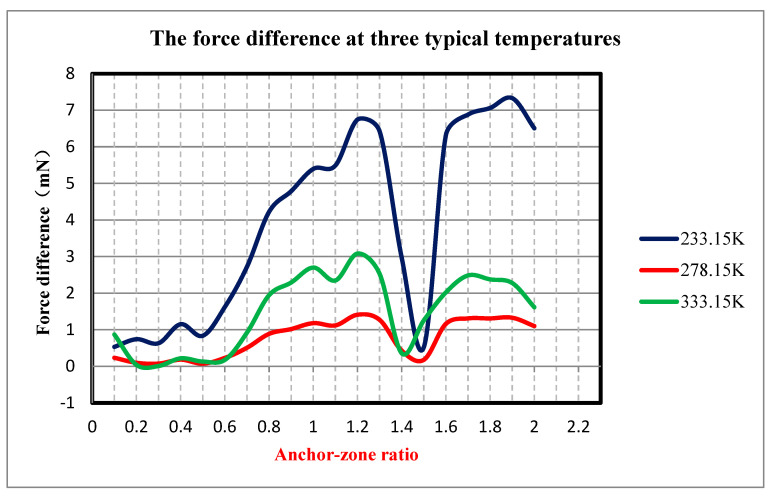
Diagram of anchor zone force difference under different anchorage zone ratios.

With the size of the silicon oxide anchor area of the current MEMS accelerometer fixed, the anchor–zone ratio varies from 0.1 to 2, and the simulations are carried out under the temperature conditions of 233.15 K, 278.15 K and 333.15 K, respectively. The simulation results are shown in Figure 5, which is the relationship between the ratio of the anchor zone and the force difference at three typical temperatures. The undulation of the curve is caused by the nonlinear nature of the material in each layer. As can be seen from the figure, the variation trends of the three curves are basically the same, and the regions with anchor–zone ratios of less than 0.6 all have gentle changes. Moreover, all three curves have the best force difference when the anchor–zone ratio is less than 0.6. The minimum force difference at the temperature 233.15 K is 5.33 × 10^−4^ N with the anchor–zone ratio of 0.1, while at temperatures of 278.15 K and 333.15 K, the smallest force difference is 7.49 × 10^−5^ N and 8.31 × 10^−6^ N, respectively, with the same anchor–zone ratio of 0.3. The simulation results are presented such that the force difference of the accelerometer-sensitive structure has a great consistency with the change trend of the anchor region ratio at different temperatures. The force of the accelerometer-sensitive structure at different temperatures can be reduced by selecting an appropriate anchor–zone ratio, so as to improve the temperature performance of the accelerometer.

**Figure 6:**
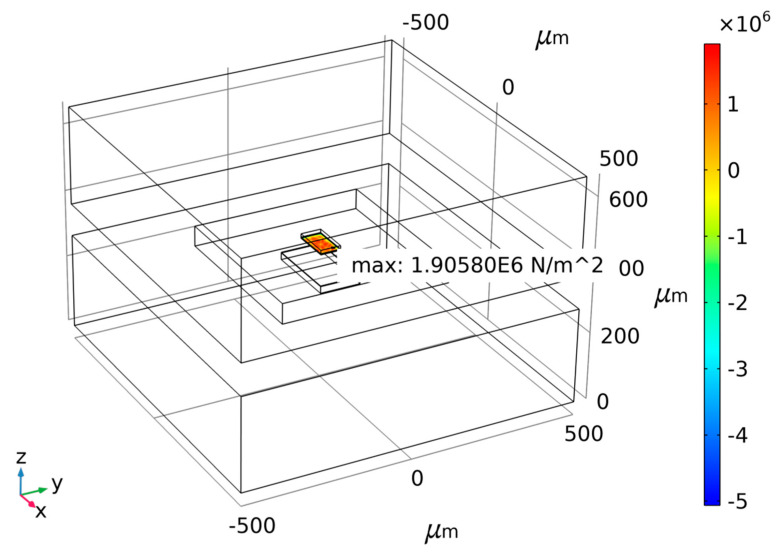
The maximum normal stress diagram of Au-Si anchor zone at 233.15 K with 0.5 anchor–zone ratio.

**Figure 7:**
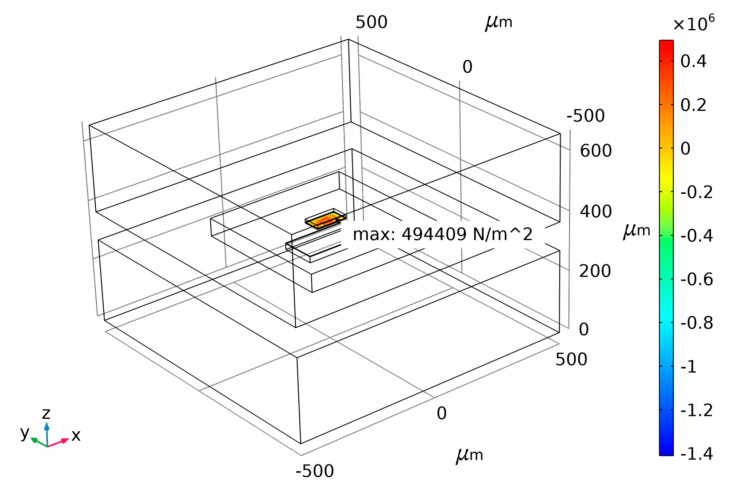
The maximum normal stress diagram of Au-Si anchor zone at 278.15 K with 0.5 anchor–zone ratio.

**Figure 8:**
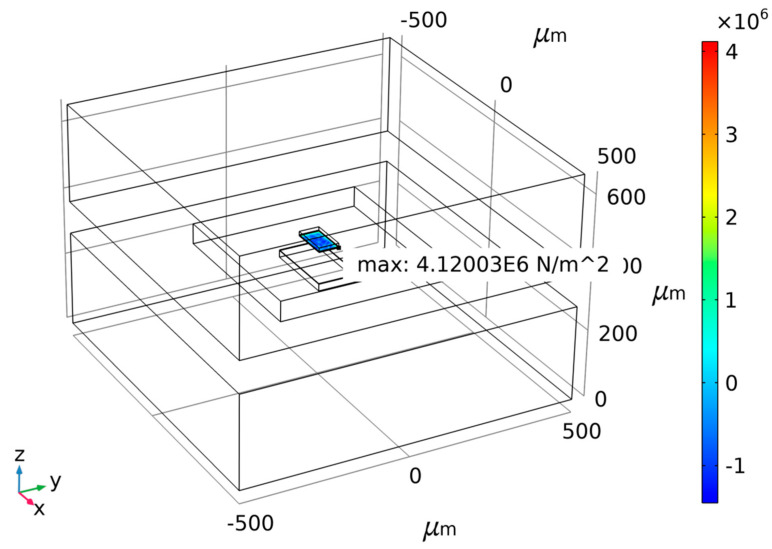
The maximum normal stress diagram of Au-Si anchor zone at 333.15 K with 0.5 anchor–zone ratio.

Taking the risks involved in actual MEMS fabricating into consideration, a small anchor area means small bonding strength. To ensure the rationality of the design and process of the MEMS accelerometer, the anchor–zone ratio of 0.1 and 0.3 is considered poor. Thus, two ratios of 0.5 and 1.5 are chosen according to the force difference diagram as shown above for different anchor–zone ratios. Therefore, the force difference fluctuation of the anchor region ratio near 1.5 is too large, and it is easy to cause a large difference because of a micromachining error. Thus, the anchor–zone ratio of 0.5 is selected as the optimal value, which ensures that the structural force is relatively small within a certain process error range. Figure 6, Figure 7 and Figure 8 show the maximum normal stress diagram of the Au-Si anchor zone at 233.15 K, 278.15 K and 333.15 K, respectively, with a 0.5 anchor–zone ratio. Figure 6, Figure 7 and Figure 8 show the maximum normal stress diagram of the Au-Si anchor zone at 233.15 K, 278.15 K and 333.15 K, respectively, with a 0.5 anchor–zone ratio.

## 5. Design Verification

**Figure 9:**
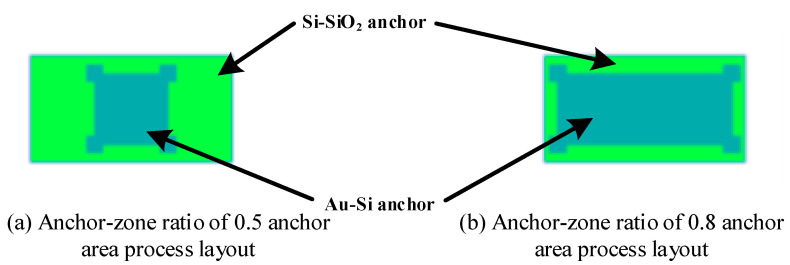
Anchor layout control group design drawing.

In order to verify the above theory, accelerometers with a 0.5 and 0.8 anchor–zone ratio were designed. The structure layout of the anchor area is shown in Figure 9. The anchor area of the Au-Si layer with an anchor–zone ratio of 0.5, as shown on the left, with an anchor area of 80 μm × 80 μm, compared with an anchor–zone ratio of 0.8 with an anchor area of 190 μm × 80 μm, as shown on the right.

**Figure 10:**
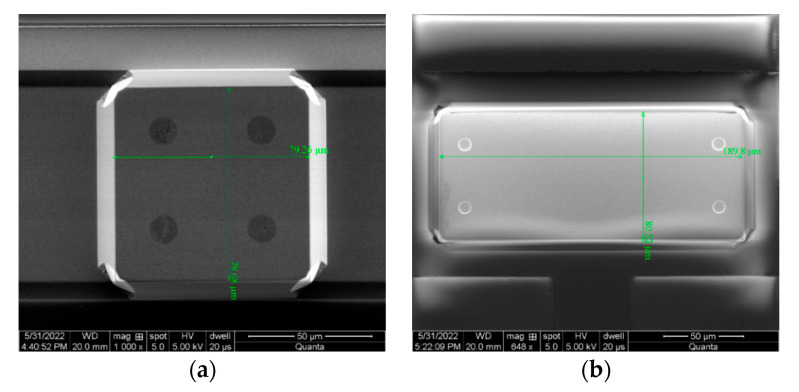
Two types of anchor SEM diagrams processed. (**a**) Anchor–zone ratio 0.5. (**b**) Anchor–zone ratio 0.8.

**Figure 11:**
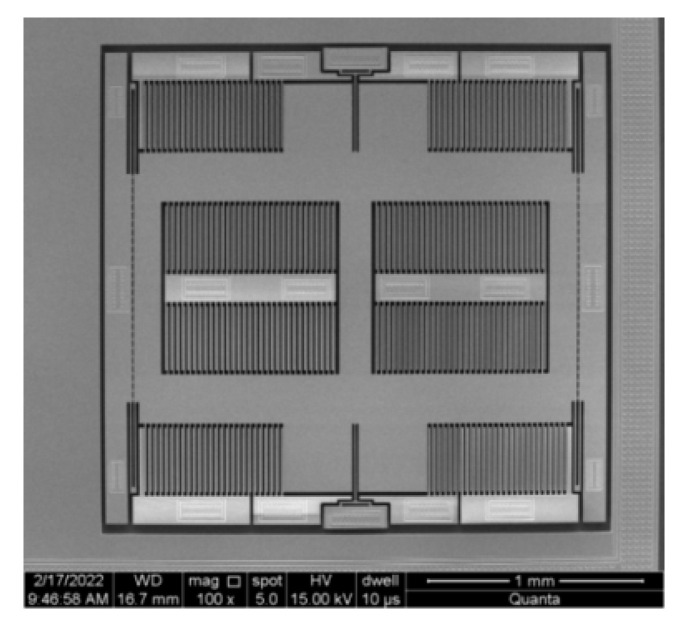
MEMS accelerometer SEM diagram of sensitive structural layers.

**Figure 12:**
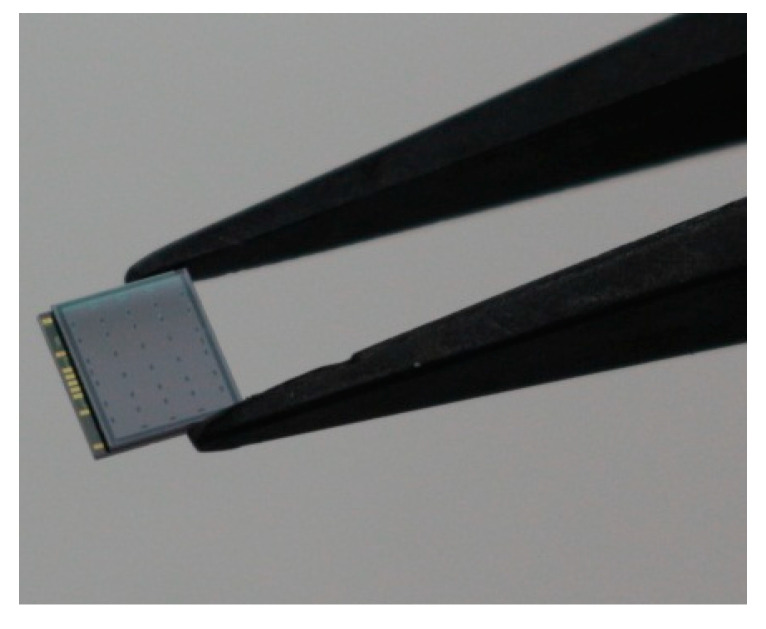
MEMS accelerometer structural chip photo.

**Figure 13:**
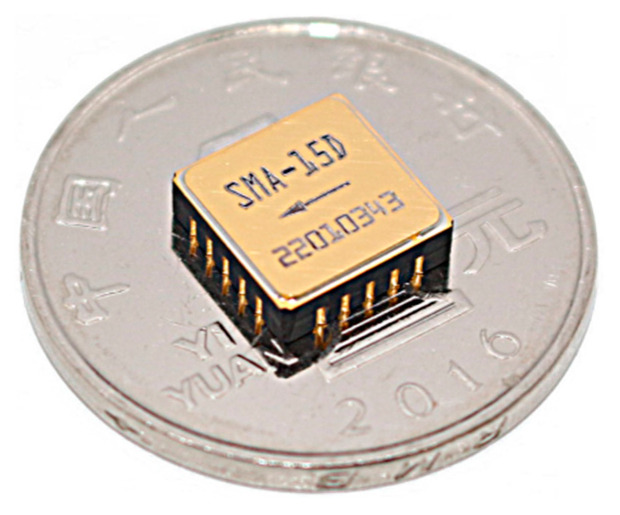
Accelerometer product photo.

Both structures were processed using the standard unit, all-silicon wafer level packaging process, and their sensitive structures are identical. The two anchor structures obtained after processing are shown in Figure 10. The sensitive structures of the two accelerometers are exactly the same, except that the anchor–zone ratio is different. Figure 11 shows the SEM image of the sensitive structure layer of the MEMS accelerometer. Figure 12 is a picture of the chip after wafer level packaging, and Figure 13 is a picture of the final packaged MEMS accelerometer product.

## 6. Experimental Test

**Figure 14:**
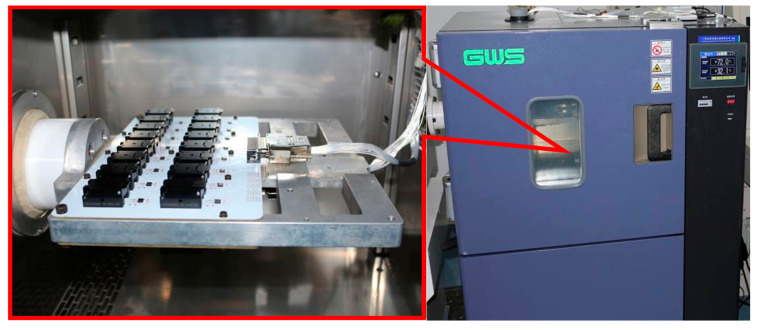
Accelerometer temperature performance test system.

**Figure 15:**
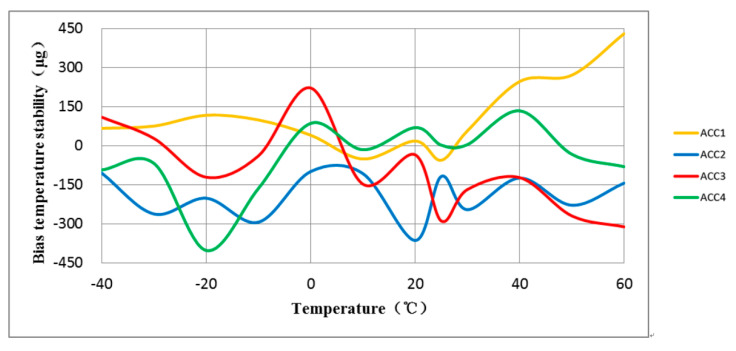
The average bias stability of the four accelerometers with an anchor-zone ratio of 0.8 was 133 μg.

**Figure 16:**
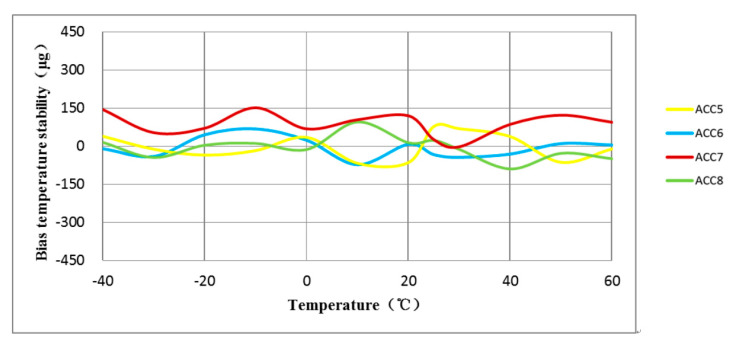
The average bias stability of the four accelerometers with an anchor-zone ratio of 0.5 was 46 μg.

**Figure 17:**
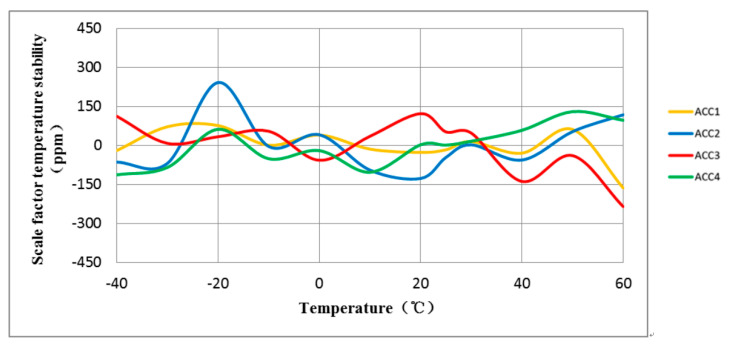
The average scale factor stability of the four accelerometers with an anchor-zone ratio of 0.8 was 87 ppm.

**Figure 18:**
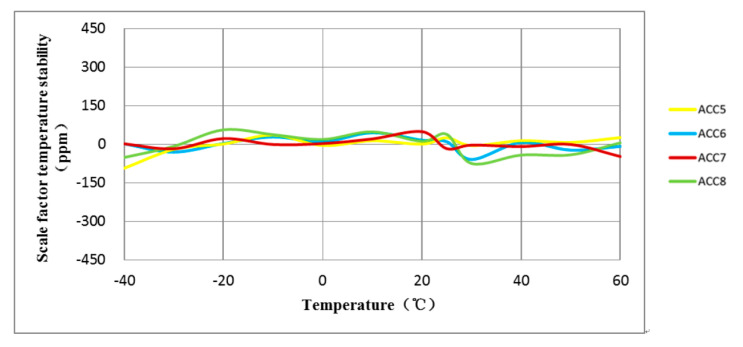
The average scale factor stability of the four accelerometers with an anchor-zone ratio of 0.5 was 32 ppm.

The experiment involved testing the full-temperature performance of four MEMS accelerometers with anchor–zone ratios of 0.5 and 0.8. The accelerometer temperature performance test system is shown in Figure 14. Results from the two groups of eight accelerometers are presented in Figure 15, Figure 16, Figure 17 and Figure 18. It can be observed that for the anchor–zone ratio of 0.8, the average zero-bias stability for acceleration is 133 μg, and the average coefficient stability for full-temperature scale is 87 ppm. On the other hand, for the anchor–zone ratio of 0.5, the average zero-deviation stability for acceleration is 46 μg, and the average full-temperature scale factor stability is 32 ppm. Compared with the two schemes, the zero-offset full-temperature performance of the scheme with an anchor–zone ratio of 0.5 is more outstanding, which has a 34.6% improvement in zero-bias stability and a 36.8% improvement in scale factor stability. The results of the comparison experiment demonstrate the effectiveness of the stress cancellation method in the anchor area of the MEMS accelerometer.

## 7. Conclusions

The simulation results illustrate that when the anchor–zone ratio of the accelerometer is reduced to 0.5, the stress in the anchor zone is significantly reduced, which improves the full-temperature performance of the accelerometer. Experiments show that if the anchor–zone ratio of the accelerometer is reduced from 0.8 to 0.5, the zero-bias full-temperature stability of the accelerometer is reduced from 133 μg to 46 μg, the scale factor full-temperature stability is reduced from 87 ppm to 32 ppm, the zero-bias full-temperature stability has a 34.6% improvement, and the scale factor full-temperature stability has a 36.8% improvement. The MEMS accelerometer has the characteristics of small size, light weight and low cost. The thermal expansion coefficient of each layer material is inconsistent. Thus, thermal stress will be generated due to the effects of temperature, which will affect the total temperature performance of the accelerometer. In order to reduce the influence of thermal stress on the multilayer structure, this paper presents a method of stress cancellation in the anchor zone, in order to reduce the internal thermal stress of structure. The experimental results show that when the anchor–zone ratio decreases from 0.8 to 0.5, the zero-offset stability of the MEMS accelerometer has been improved to 34.6%, and the full-temperature scale factor stability has been improved to 36.8%. The whole temperature performance of accelerometer is improved.

## Figures and Tables

**Table 1:** Accelerometer Layer Material Attribute Table.

Name	Silicon	Silicon Dioxide	Au	Unit
Coefficient of thermal expansion (CTE)	2.60 × 10^−6^	0.5 × 10^−6^	14.2 × 10^−6^	1/K
Constant pressure heat capacity	700	730	129	J/(kg·K)
Density	2329	2200	19,300	kg/m^3^
Thermal conductivity	130	1.4	317	W/(m·K)
Young’s modulus	1.69 × 10^11^	7.00 × 10^10^	7.00 × 10^10^	Pa
Poisson’s ratio	0.28	0.17	0.44	1

## Data Availability

Not applicable.
